# Nanoscale investigation of bone tissue near lacunae of trabecular bone in type-1 diabetic postmenopausal women

**DOI:** 10.1093/jbmrpl/ziag139

**Published:** 2026-08-19

**Authors:** Maxwyll McConnell, Wen Qian, Luke Schwaninger, Eleftherios P Paschalis, Laura A Graeff-Armas, Susan P Bare, Joseph A Turner, Sam A Dubrow, Joan M Lappe, Robert R Recker, Mohammed P Akhter

**Affiliations:** University of Nebraska-Lincoln, Lincoln, NE 68588-0526, United States; University of Nebraska-Lincoln, Lincoln, NE 68588-0526, United States; University of Nebraska-Lincoln, Lincoln, NE 68588-0526, United States; Ludwig Boltzmann Institute of Osteology, A-1140 Vienna, Austria; University of Nebraska Medical Center, Omaha, NE 68198, United States; Osteoporosis Research Center, Creighton University, Omaha, NE 68122, United States; University of Nebraska-Lincoln, Lincoln, NE 68588-0526, United States; Osteoporosis Research Center, Creighton University, Omaha, NE 68122, United States; Osteoporosis Research Center, Creighton University, Omaha, NE 68122, United States; Osteoporosis Research Center, Creighton University, Omaha, NE 68122, United States; Osteoporosis Research Center, Creighton University, Omaha, NE 68122, United States

**Keywords:** trabecular bone, diabetes, postmenopausal women, nanoindentation, lacunae, nano-IR

## Abstract

The goal of this study is to investigate the causes of skeletal fragility in type 1 diabetic (T1D) women. We hypothesize that bone fragility in diabetic individuals is partly due to changes in mineral and/or intrinsic material properties in the osteocyte lacunar/peri-lacunar regions of bone tissue. Studies of bone material properties in T1D are limited, and BMD alone does not explain the elevated fracture risk in T1D women (Cases). Innovative instruments with nanoscale resolution, including a laser scanning microscope, an atomic force microscope that is integrated with infrared spectroscopy, and a nanoindenter, were used for the characterization of the material properties surrounding osteocyte lacunae. In trabecular bone tissue, the compositional Mineral Matrix Area (MMA) and MMP ratios (for both near- & far-lacunae), along with material strength variables (Modulus, Hardness for both near- & far-lacunae), were lower in T1D compared to Controls. While the material compositional ratio of mineral maturity, crystallinity, and area was higher, the mineral maturity, crystallinity peak, and hardness tended to be higher in “lacunae-near” than “lacunae-far” for all the biopsies. Furthermore, the higher mineral matrix (MMA) and material strength (modulus, hardness) in peri-lacunar regions of Controls may suggest its increased brittleness or differences in properties as compared to T1D. We postulate that the difference between Cases (T1D) and Controls in modulus and hardness could be due to the variation in mineral exchange (diffusion) rate within the perilacunar space.

## Introduction

It is well understood that both the material properties and the mineral quantity of bone tissue determine bone strength.^1^^,^^2^ This is especially evident in the case of diabetic patients, where many studies have indicated that individuals with type 1 diabetes are at a higher risk of skeletal fractures compared to non-diabetics; however, the BMD alone could not predict the fracture occurrence of T1D patients.^3–15^ While it is hypothesized that hyperglycemia, hypoinsulinemia, autoimmune inflammation, higher advanced glycation end-product (AGE) content, low levels of IGF-1, and vitamin D are responsible for bone tissue differences, the precise mechanism(s) responsible for the skeletal fragility in T1Ds is not yet known.^3^^,^^8^^,^^12^^,^^13^^,^^16^ Although accumulation of advanced glycation end products has been proposed as the underlying pathological mechanism leading to fracture, a recent review paper recommends additional investigation because there is no clear consensus whether the observed elevated AGEs content in patients with T1D is happenstance or involved in the mechanism resulting in fragility fracture occurrence (rather than risk).^17^

Within trabecular bone tissue, osteocytes play a major role in mechanotransduction and are central regulators of bone remodeling and calcium homeostasis [Parfitt, 1976].^18–24^ The areas containing osteocytes are small oval-shaped spaces called “lacunae.” Furthermore, canaliculi are small channels that link adjacent lacunae together and allow for the transportation of nutrients and waste products through bone tissue. This arrangement suggests that the density of lacunae with a greater percentage of mineralized osteocyte lacunae contributes to a higher bone tissue hardness.^1^^,^^10^^,^^19^^,^^25^ Therefore, it is hypothesized that the physiological response to diabetes and its treatment protocols could alter the mineralization of osteocyte lacunae or the density of the lacunae altogether, thus, affecting the intrinsic material and compositional properties of bone tissue.^9^^,^^10^^,^^26^^,^^27^

Biological variability confounds studies on compositional and mechanical properties at the microscopic level (examination of bone tissue at specific microanatomical locations such as specific tissue ages, cement lines, osteocytes, etc.). This is even more important for studies attempting to decipher the relationship between bone compositional and mechanical properties at the micron level.

Fourier transform infrared spectroscopy has been used to establish bone material quality along with compositional properties: mineral-to-matrix ratio, crystallinity (ie, mineral crystal size), carbonate-to-phosphate ratio (ie, mineral maturity and crystallinity ratio), and collagen maturity (ie, the level of enzymatic cross-linking).^2^^,^^8^^,^^12–15^^,^^19^^,^^28^^,^^29^ The atomic force microscope that is integrated with infrared spectroscopy (AFM-IR) provides chemical analysis with nanoscale spatial resolution that is far better than conventional optical diffraction techniques. AFM-IR uses an AFM contact probe to detect the local thermal expansion in a sample, which results from absorption of infrared radiation during a measurement. Such an approach can also be used to map the chemical response spatially in bone tissue.^15^^,^^18^^,^^30^^,^^31^

We hypothesized that peri-lacunar regions in healthy controls would demonstrate higher mineral-to-matrix ratios and greater intrinsic mechanical strength compared to T1D samples, and that these spatial compositional properties would be diminished in T1D bone. In this manuscript, we combine three different approaches, including AFM-IR, nanoindentation, and confocal laser scanning microscope (LSM), to characterize osteocyte lacunar and peri-lacunar properties at the micron scale, in bone biopsies from postmenopausal women with long-term T1D (Cases, *n* = 8) and appropriate age-, BMD-, and clinically-matched postmenopausal women (Controls; *n* = 5). To the best of our knowledge, this is the first study to investigate the causes of T1D-related skeletal fragility in postmenopausal women resulting from the compositional/material properties of peri-lacunar space.

## Materials and methods

### Position registry

The mineral organization and mechanical properties in the peri-lacunar region were quantified using three different experimental techniques, including LSM/AFM-IR/nanoindentation, all with nanoscale spatial resolution. All the regions of interest (ROI) were within ~25 μm of the center of each lacuna. Each lacuna was identified/located when transferred between instruments (LSM to AFM-IR, to IIT), so that the data from various measurements could be correlated spatially. First, each lacuna of interest was identified with LSM within a bone biopsy sample. The location/position of each lacuna was recorded relative to the specific shape of the central canal as well as the surrounding cement lines as fiducial markers. Next, the mineral organization measurements were made using AFM-IR. Using the fiducial markers, each lacuna was identified with an optical microscope, and the probe was positioned accordingly in the peri-lacunar space. Localized nanoIR spectra were acquired at several positions in this region and relevant nanoIR absorption peaks were identified. Finally, the samples were tested with nanoindentation for their nanoscale mechanical properties of elastic modulus and hardness in the peri-lacunar region using fiducial markers to identify the target lacuna in order to position the indenter tip in the regions of interest.

### Patients and bone sample preparation

The Control group (*N* = 5) consisted of postmenopausal patients, while the T1D Case group (*N* = 8) of age- and BMD-matched postmenopausal, non-fracturing type 1 diabetics. All participants were Caucasian, >50 yr old, and 5 yr past the onset of menopause. T1D patients had diabetes for 10-30 yr. All of the subjects, Case and Control, were free of diagnoses affecting bone metabolism (other than diabetes in the T1D group), and had dual-energy x-ray absorptiometry (DXA) T-scores at the TH and LS between −1.0 and −2.5. Each Control subject was matched to the T1D cases with the following criteria: (1) DXA measures (BMD, gm/cm) were within +/− 10% in the spine and hip, (2) BMI was within +/− 10%, and (3) Age within +/− 5 yr. All bone biopsies were from the transiliac crest and obtained using standard procedures described elsewhere.^12^^,^^32^

All analyzed biopsies in the present report were embedded in low-viscosity, quick-setting EpoThin plastic material (Buehler) as described elsewhere.^12^^,^^33^ To prepare the bone samples, a longitudinal section of 250 μm in thickness was cut from each bone biopsy. The longitudinal section’s surface of the bone was polished with varying degrees of sandpaper and buffing solutions. The polishing grit order was 600/P1200 and 1200/P2500 silicon carbide sandpaper and a 1 μm alumina slurry buff to finish.

### Localized nanoIR spectrum for mineral and matrix characterization

**Figure 1:**
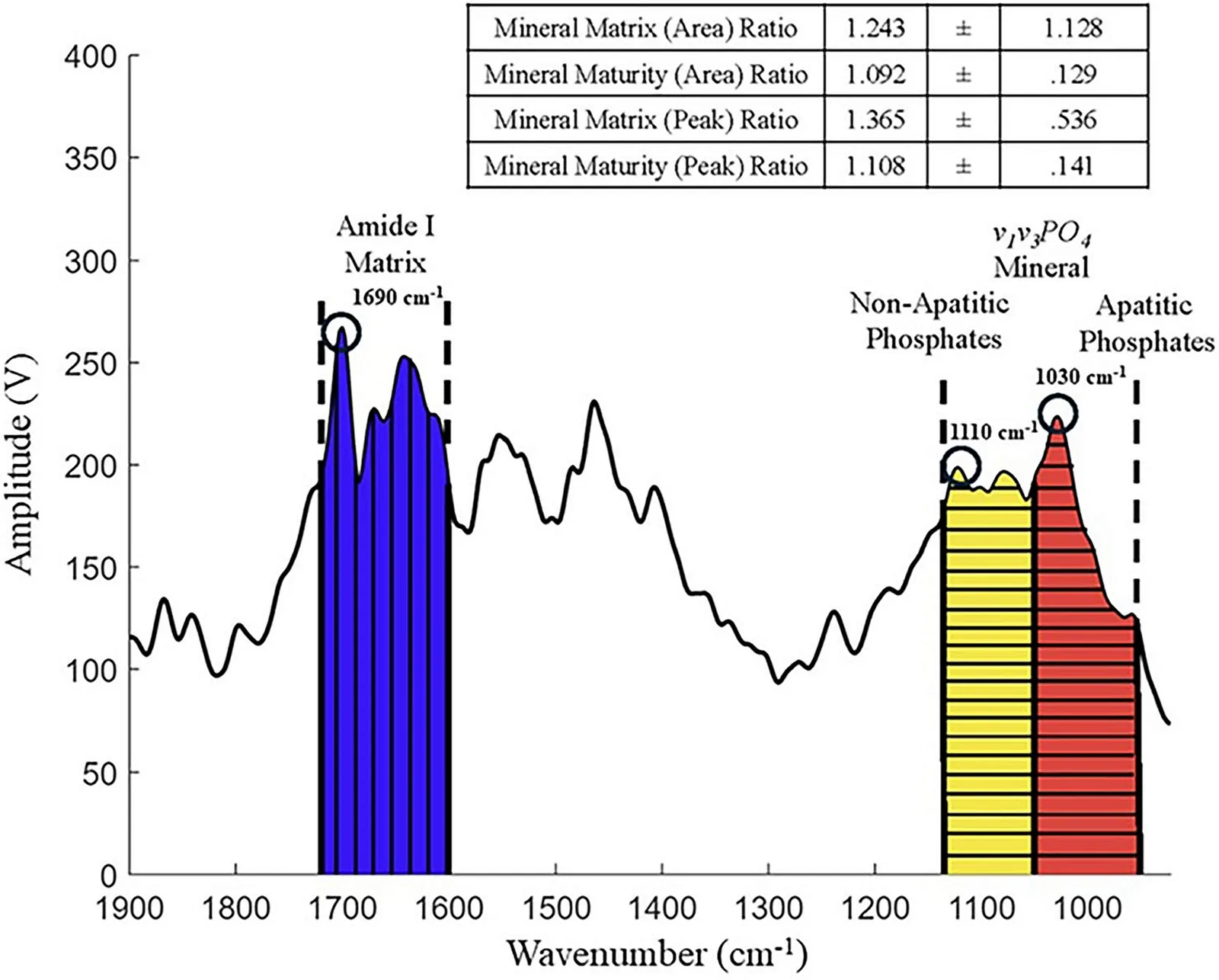
Schematic illustration (example) of area ratio and peak ratio for a localized infrared spectrum of a Case (T1D) bone biopsy specimen. (Mean+/−SD).

For bone compositional analysis, mineral and matrix characterization via AFM-IR was performed. A commercial nanoIR2 atomic force microscope (Anasys Instruments, Inc.) supplemented with a tunable infrared quantum cascade laser, QCL (Daylight Solutions MIRcat), was used to image topography and measure localized nanoIR spectra, as well as to create chemical IR maps at a constant wavelength. Contact mode nIR2 probes (Model: PR-EX-nIR2, Anasys Instruments) with a resonance frequency of 13 ± 4 kHz and spring constant of 0.07-0.4 N/m were used. The AFM-IR measurements were accomplished by coupling an AFM with a pulsed tunable IR source, which has a pulse length of ∼10 ns and can cover a broad range of mid-IR regions. The light from the IR laser was focused onto the surface region near the tip-sample contact area. When the sample absorbed the light, a rapid heating/expansion of the sample occurred, which created an impulsive load onto the AFM cantilever tip that induced oscillation. The amplitude of the cantilever oscillation was proportional to the sample IR absorption coefficient. Spectra were acquired with two perpendicular directions of infrared laser light. To enable visualization and comparison of spectral shapes and peak intensity ratios, all nanoIR spectra were normalized by scaling their peak amplitudes relative to the maximum intensity within each individual spectrum. Because residual embedding medium may remain within the lacunae, a background nanoIR spectrum was first collected from the exposed surface of a lacuna to capture its spectral contribution. This background spectrum was then automatically subtracted from the nanoIR spectra collected in adjacent bone regions, ensuring that the resulting data accurately reflect the composition of the bone matrix itself. Spectra were acquired over a range of 1900 to 912 cm^−1^. Each spectrum was acquired two times, automatically averaged for better signal-to-noise ratio, and the background noise was subtracted using the instrument software. The individual spectra were fit with Gaussian functions using a customized MATLAB code (MathWorks, Inc.). The mineral-to-matrix ratio was calculated by dividing the area of the *v_1_,v_3_* phosphate peak (1140-950 cm^−1^) by the area of the amide I peak (1720-1600 cm^−1^).^8^^,^^34^ The mineral maturity ratio was calculated by dividing the apatitic phosphate peak area by the non-apatitic phosphate peak area (1030 cm^−1^/1110 cm^−1^).^8^^,^^34^ The 1030 cm^−1^/1110 cm^−1^ area ratio was chosen because the 1020 cm^−1^ line is not clearly resolved on raw spectra and thus is not identifiable compared with the 1110 cm^−1^ line. The definitions used for the calculations are shown schematically in Figure 1. These values were used for comparisons between the Controls and Cases. The “lacunae-near” position is defined as within 5 μm from the lacunar edge. Based on the lacunar size variation, T the “lacunae-far” position is defined as between 15-25 μm from the lacunar edge. These locations were chosen to ensure that the “lacunae-near” positions would be mineralized bone in the Control, allowing for consistent comparisons with the Case group. For each lacuna, we chose 7 to 9 points around “lacuna-near,” and another 7 to 9 points around “lacunae-far.” A total of 650 nanoIR spectra were collected on a total of 41 lacunae, 12 lacunae from Control samples, and 29 lacunae from Case samples. On average, 2-4 lacunae were analyzed per biopsy specimen depending on availability and image quality. All analyzed lacunae were located within trabecular bone matrix and were selected away from trabecular surfaces to minimize potential effects of active remodeling. nanoIR Spectra were smoothed in Analysis Studio and plotted in a MATLAB program to fit curves for peak and area values. Bone tissue compositional ratios (Mineral-to-Matrix Area Ratio [MMA], Mineral-Matrix-Peak Ratio [MMP], Mineral Maturity and Crystallinity Area Ratio [MMCA], and Mineral Maturity and Crystallinity Peak Ratio [MMCP]) were calculated with regard to their location (near or far) (Table 1) and analyzed for their difference between controls and T1D cases (Table 2) based on previously published work.^8^^,^^18^^,^^34^

**Table 1:** Localized infrared property of lacunae with regards to their location (near or far) in Control and Case (T1D) pairs.

**Type**	**Mineral matrix area (MMA) Ratio**	**Mineral maturity crystallinity area (MMCA) ratio**	**Mineral matrix peak (MMP) ratio**	**Mineral maturity crystallinity peak (MMCP) ratio**
**Control lacunae-near**	2.856 ± 0.196	0.807 ± 0.091	3.484 ± 0.259	0.964 ± 0.174
**Control lacunae-far**	2.866 ± 0.211	0.846 ± 0.041	3.496 ± 0.339	1.055 ± 0.057
**Case lacunae-near**	1.327 ± 0.211	1.011 ± 0.090	1.769 ± 0.248	1.211 ± 0.187
**Case lacunae-far**	1.362 ± 0.270	.979 ± 0.055	1.703 ± 0.300	1.143 ± 0.134

These data (Table 1) reflect variability or heterogeneity within a pair of samples (Case and Control) showing mineral/matrix (MM) and mineral maturity and crystallinity (MMC) distribution around lacunae.

(Mean ± SD).

**Table 2:** Bone tissue’s compositional properties around peri-lacunar space.

	**Case (T1D)**	**Control**
**Lacunae-near & far**		
**Mineral matrix area (MMA) ratio**	1.706 ± 1.200^a^	2.134 ± 0.839
**Mineral maturity crystallinity area (MMCA) ratio**	1.099 ± 0.177	1.095 ± 0.235
**Mineral matrix peak (MMP) ratio**	1.888 ± 0.849^a^	2.297 ± 0.839
**Mineral maturity crystallinity peak (MMCP) ratio**	1.128 ± 0.194^a^	1.087 ± 0.159
**Lacunae-near**		
**Mineral matrix area (MMA) ratio**	1.738 ± 1.386^a^	2.117 ± .774
**Mineral maturity crystallinity area (MMCA) ratio**	1.117 ± 0.207	1.125 ± 0.278
**Mineral matrix peak (MMP) ratio**	1.893 ± .895^a^	2.264 ± .890
**Mineral maturity crystallinity peak (MMCP) ratio**	1.130 ± .201^b^	1.099 ± .177
**Lacunae-far**		
**Mineral matrix area (MMA) ratio**	1.673 ± .984^a^	2.151 ± .900
**Mineral maturity crystallinity area (MMCA) ratio**	1.082 ± .138^b^	1.067 ± .182
**Mineral matrix peak (MMP) ratio**	1.883 ± .803^a^	2.328 ± .790
**Mineral maturity crystallinity peak (MMCP) ratio**	1.125 ± .188^a^	1.076 ± .140

Summary of the group level comparisons across all samples.

Mean ± SD.

^a^Difference between Case and Controls, *p* < .05.

^b^Difference between Case and Controls, *p* < .5.

### Nanoindentation testing of mechanical properties

The localized mechanical properties (modulus and hardness) of the peri-lacunar region were measured using a Hysitron TI 950 Triboindenter through quasistatic nanoindentation.^12^^,^^18^^,^^28^^,^^35–39^ A Berkovich (three-sided pyramid) diamond tip was mounted on a transducer that allowed for displacements in the z-direction. The bone sample was mounted on a scanner that allowed for motion in the x/y-plane that was perpendicular to the tip axis. This combination allowed the topography of the sample surface to be mapped, using the scanning probe microscope (SPM) mode, and force-displacement measurements to be made using the same tip. Based on the SPM image, the tip was positioned on the peri-lacunar region to quantify mechanical differences with respect to position. During an indentation measurement, the tip was pressed into the sample such that the resulting force-displacement behavior was quantified. Multiple indentations were made with a target force of 6 mN (milliNewtons) at a constant loading rate of 400 μN/s (microNewtons per second).^18^^,^^33^ The indentation procedure included a linear loading ramp of 15 s, a holding period of 10 s at the maximum load, and a linear unloading ramp of 15 s. The load-displacement data from each indentation were used to calculate the reduced indentation modulus (Er) and hardness (H) for the tissue. Mechanical measurements of modulus and hardness were made with ~10 μm spacing, in order to identify differences in the volume fraction of minerals near the lacunae. Poisson’s ratio of 0.3 was assumed for bone tissue in calculations for the analysis.^18^ The Oliver-Pharr method was used to extract the indentation modulus.^37^ A total of 310 nanoindentation results were collected on a total of 40 lacunae, 12 lacunae from Control samples, and 28 lacunae from Case samples.

### Compositional vs mechanical properties

The slopes between the compositional properties were analyzed for significant relationships between various compositional and mechanical properties. For instance, at measured sites of lacune, slopes we analyzed between eight pairs (MMA vs [Er & H], MMP vs [Er, & H], MMCA vs [Er & H], and MMCP vs [Er & H]).

### Statistics analysis

Both nanoIR spectra and Hysitron nanoindentation statistics were analyzed using IBM SPSS-v23. For comparing “lacunae-near” and “lacunae-far” values for a set of samples, a paired *t*-test was used. For comparison between Case and Control samples, an unpaired *t*-test was used. Table 1 and Table 3 show one representative matched Case–Control pair to illustrate local variability, whereas Table 2 and Table 4 summarize group-level comparisons.

**Table 3:** The variability of modulus and hardness values from 4 indents for each lacunar location (near & far) between one pair of Case (T1D) and Control.

	**Case (T1D)**		**Control**	
	Modulus (GPa)	Hardness (GPa)	Modulus (GPa)	Hardness (GPa)
**Lacunae-near**	10.948 ± 0.725	0.618 ± 0.077	19.456 ± 2.292	0.8095 ± 0.182
**Lacunae-far**	9.992 ± 0.603	0.527 ± 0.054	19.148 ± 0.187	0.7185 ± 0.080

These data present nanoindentation results from a representative matched Case–control pair to demonstrate local variability from four indents at each lacunar location.

**Table 4:** Modulus and hardness between “Case” and “Control” via unpaired *t*-test statistical analysis.

	**Case (T1D)**	**Control**
**Near & far**		
**Modulus (Gpa)**	15.998 ± 4.450^a^	17.875 ± 4.283
**Hardness (Gpa)**	0.5135 ± 0.2109^a^	0.6052 ± 0.2625
**Lacunae-near**		
**Modulus (Gpa)**	15.021 ± 6.367^a^	17.766 ± 4.437
**Hardness (Gpa)**	0.5569 ± 0.2550^a^	0.6111 ± 0.2700
**Lacunae-far**		
**Modulus (Gpa)**	14.92 ± 6.098^a^	18.134 ± 4.087
**Hardness (Gpa)**	0.5467 ± 0.2394^a^	0.6055 ± 0.2575

The table represents summary of the group level comparisons across all samples.

Mean ± SD.

^a^Difference between Case and Controls, *p* < .05.

## Results

**Figure 2:**
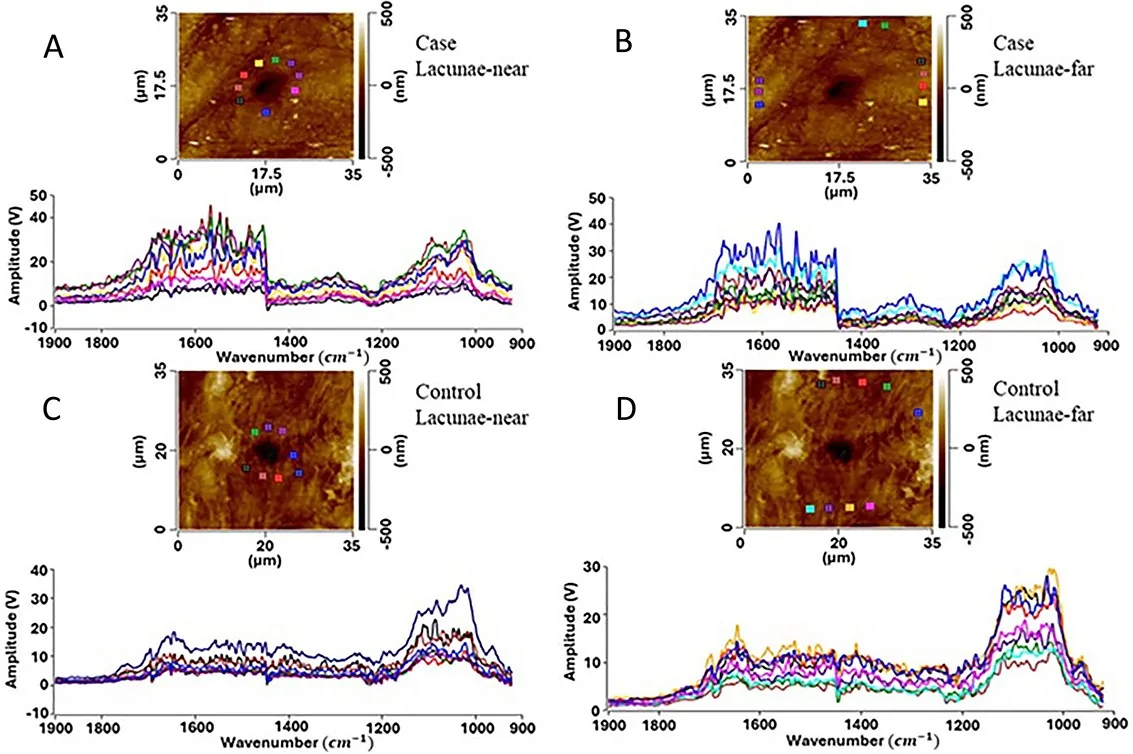
Comparison of localized IR spectrum between “lacunae-near” and “lacunae-far” for Case (A and B) and Control (C and D) samples respectively. Color of each spectrum corresponds to the local perilacunar region analyzed (shown by square symbols).

Figure 2 shows the comparison of localized IR spectra between “lacunae-far” and “lacunae-near” for one pair of Case and Control samples, respectively. A total of 14 localized IR spectra were collected, 7 around each location site “lacunae-near” and “lacunae-far,” within the IR spectra range from 922 to 1900 cm^−1^. The IR spectra were fit, and the MMA ratio, MMP ratio, MMCA ratio, MMCP ratio of distance-related lacunae were extracted (summarized in Table 2).^8^^,^^34^ MMA and MMP ratios (both near & far lacunae) were lower in T1D compared to Controls (Table 2). Furthermore, while material compositional ratio of mineral maturity crystallinity area (MMCA) was higher, the mineral maturity crystallinity peak (MMCP) tended to be higher in “lacunae-near” than “lacunae-far” for all the biopsies.

**Figure 3:**
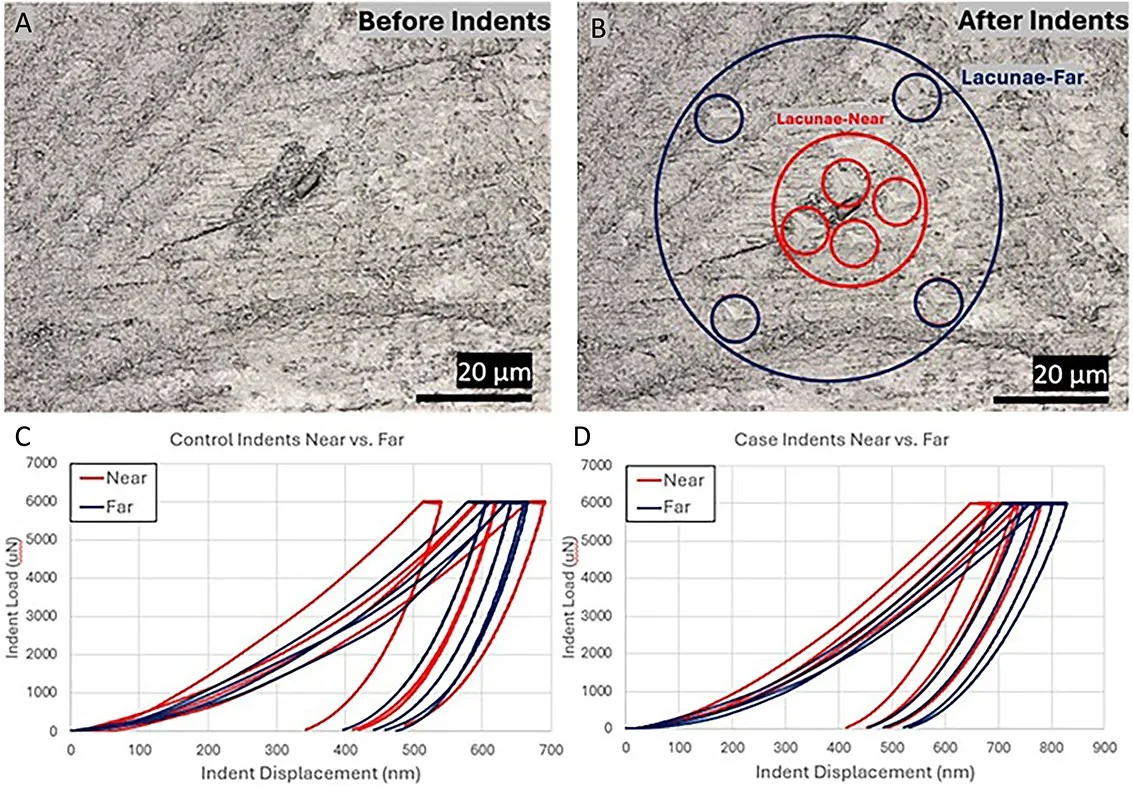
(A and B) Laser scanning images before and after indentation, a total of 8 indents around the lacuna, four indents within 5 μm of the lacuna, four indents located from 15-25 μm; (C and D) indentation curves showing force (μm) vs depth (nm) of indents around a Case (T1D) and Control sample lacuna. Each sample received 4 indents located within 5 μm of the lacuna and 4 located from 15 to 25 μm, for a total of 8 Case and 8 Control curves.

The location of “lacunae-near” was within the 5 μm and “lacunae-far” was within the 15 μm distance from each lacunar edge. The distance between “near” and “far” was around 10 μm. LSM was utilized to find the lacunar sites that were indented and take a high-resolution laser scanning image of the location of the indents to ensure they were placed correctly around the lacunae, as shown in Figure 3A and B. Eight typical nanoindentation curves on both “lacunae-near” and “lacunae-far” are shown in Figure 3C and D and the comparison of modulus and hardness values between one pair of Case and Control are shown in Table 3.

The comparisons of modulus and hardness between “Case” and “Control” via unpaired *t*-test statistical analysis are shown in Table 4; It was observed that there was a statistically significant (*p* < .05) difference between T1D and Control for modulus and hardness values. Both the modulus and Hardness were lower in T1D (cases) compared to controls (Table 4). However, when comparing “Lacunae-near” to “Lacunae-far” values for all biopsies (combined groups), there is non-significant difference (*p* < .5) in hardness values. The “Lacunae-near” hardness value tends to be higher in these groups, representing relatively stronger bone properties closer to the lacuna. Furthermore, the SDs in the T1D tended to be greater for few of the measured variables. Although heterogeneity has been associated with good quality, an overly elevated variation may also be detrimental (eg, osteogenesis imperfecta) to bone structure and strength, thus may be partially responsible for the decrease in strength in T1D individuals.^40^

**Figure 4:**
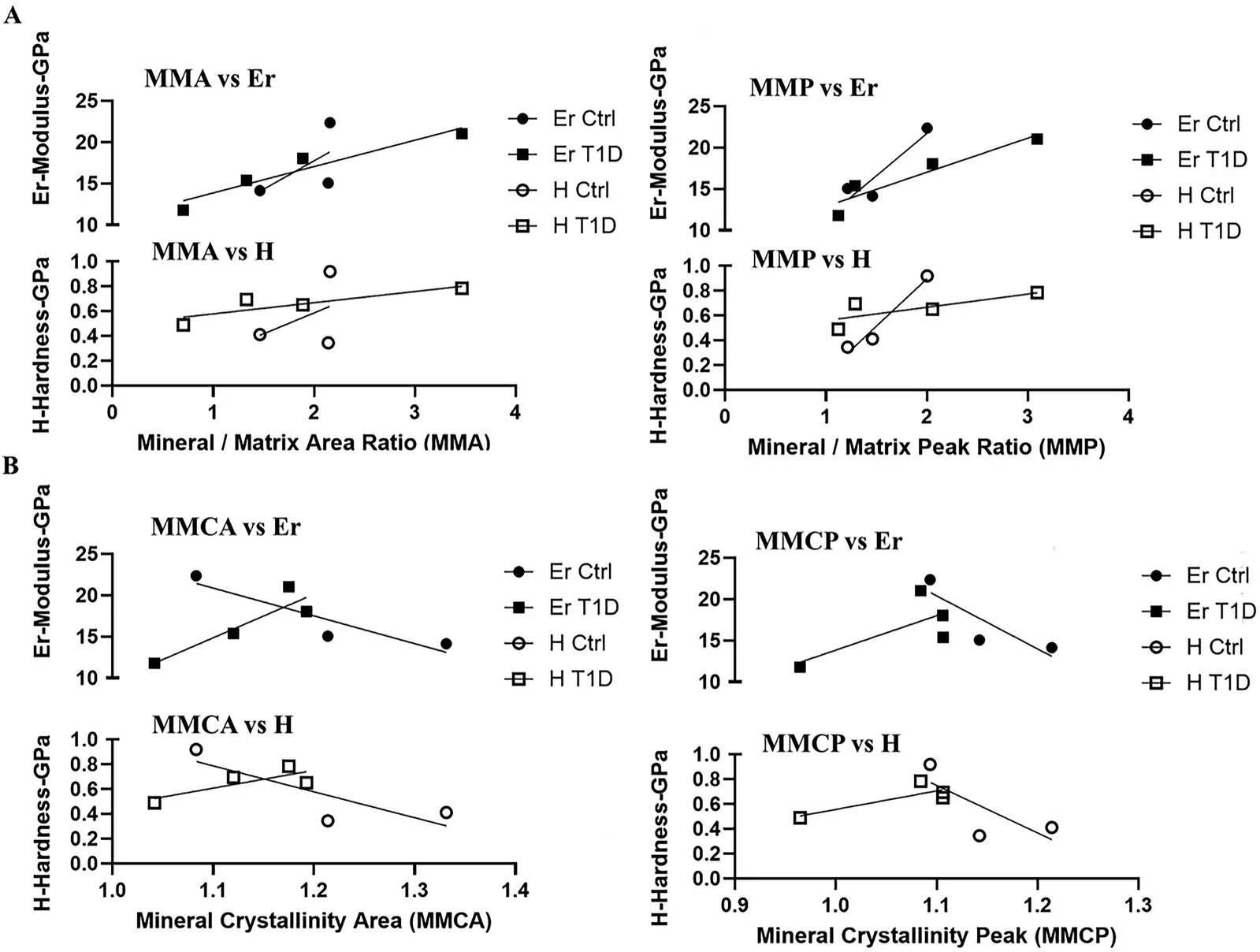
The mineral matrix compositional properties (MMA & MMP) at the lacunar site have positive slopes/relationship with both the modulus (Er) and hardness (H) (A) in both Controls and Case (T1D) patients. However, in the Controls, the mineral maturity crystallinity (MMC and MMCP) showed inverse relationship with H only (B).

The mineral matrix compositional properties (MMA & MMP) at the lacunar site have positive slopes/relationships with both the modulus (Er) and hardness (H) (Figure 4A) in both Controls and T1D patients. However, in the Controls, the mineral maturity crystallinity (MMC and MMCP) showed an inverse relationship with H only (Figure 4B). In Figure 4 plot, we matched the number of biopsies that have been subjected to both the nano-Indentation and Nano-IR to show the trends or relationships between mechanical and compositional properties in the peri lacunar areas. We performed nanoindentation first and then analyzed the compositional properties using nano-IR. These plots suggest that while hardness/modulus have a positive relation with mineral matrix (MMA & MMP), they show a negative relation with crystallinity (MMCA & MMCP).

## Discussion

We used the nanoIR technique to quantify the compositional properties at the perilacunar sites in T1D and controls. These sites are affected by skeletal conditions such as increased mineralization due to shrinking osteocyte lacunar space and diseases.^25^^,^^41^ The compositional characteristics that include both mineral-to-matrix and crystallinity ratios are important in determining the local material strength properties of bone tissue such as hardness and modulus.^12^^,^^42^ In trabecular bone tissue, the compositional (MMA and MMP for both near and far lacunae) along with material strength variables (modulus and hardness for both near- and far-lacunae) were lower in T1D compared to Controls. Osteocyte lacunar space is a dynamic region with regard to mineral changes,^41^ and therefore may also affect the overall material properties of bone tissue. The changes in both the mineral maturity and crystallinity in T1D bone tissue reflect declining values of the material strength (modulus, hardness) properties when compared with Controls. The increased MMCP ratio and the relatively modest changes in mineral maturity area ratio (MMCA) observed in T1D bone tissue were associated with lower material strength properties (modulus and hardness) compared with Controls. It is important to point out that the bone biological tissue is a complex composite that contains not only type I collagen fibrils, but also mineral and proteoglycan components.^43^ Therefore, various tools, including nano-IR, are important to understand the compositional organization of bone tissue affected by various clinical conditions such as diabetes. Previous studies have shown that diabetes alters bone matrix composition through increased AGEs accumulation, changes in collagen, and impaired osteocyte function. Our findings extend these observations by showing that compositional and mechanical property changes also occur in the osteocyte peri-lacunar region, providing additional insight into the underlying mechanisms contributing to the elevated fracture risk characterizing T1D patients.

In the present study, we found evidence that trabecular bone from T1D samples have lower hardness and modulus compared to Controls around peri-lacunar space. Furthermore, the “lacunae-near” tended to have higher hardness, which reflects its greater compositional properties as compared to the “lacunae-far in all biopsies.” Although AGEs have been implicated in deterioration of mechanical properties in T1D, their true role in bone fragility is not fully established.^17^ Our previous work^12^ suggests that the T1D samples have higher quantities of these AGEs, which likely affect the hardness farther from the lacunae and in the interstitial bone tissue. This is supported by AFM-IR data, which shows that the mineral matrix area ratio of both “lacunae-near” and “lacunae-far” in Case samples are significantly lower than Controls, but with no significant difference for mineral maturity ratio between the two groups. Similar to the current work, our published data for nanoIR and nanoindentation^18^ in biopsies from fracturing (Case) and non-fracturing (Control) postmenopausal women suggested that the Controls had greater mineral matrix and intrinsic strength properties at the near lacunar site.

Also, in our published work^12^ in biopsies from T1D and controls, the composition and strength properties in the interstitial bone (both at the cement lines and interstitial regions) were different than measured at the peri-lacunar sites here. Unlike the peri-lacunar tissue here, the interstitial bone tissue shows reduced mineral-to-matrix ratios and decreased mechanical strength properties in T1D compared to age- and BMD-matched controls. Also, the relative increase in mineral maturity crystallinity (MMCA and MMCP) at lacunae-near compared to lacunae-far sites suggests spatially dependent mineral exchange mechanisms, which are specific to lacunar voids referred to as mineral diffusion and micropetrosis,^25^^,^^41^ a different mineralization process than remodeling, which involves resorption followed by formation (in interstitial bone tissue). Furthermore, along with differences in mineral exchange (diffusion) at the peri-lacunar space, the accumulation of AGEs may also be different due to variation in the presence of collagen at the same level as expected in the interstitial bone tissue.^12^ Although interstitial bone represents previously formed bone between BMUs, it is still subjected to Perilacunar/Canalicular Remodeling (PLR), thus, its material properties are influenced by prior remodeling events as well as diffusion processes.^43^ Nevertheless, the mineral properties (maturity, crystallinity, etc.) in the lacunar region may have a similar influence on the material strength properties (hardness and modulus) of bone tissue, as reported previously.^12^ PLR diffusion-driven mineralization within a pre-existing mineralized bone matrix contributes to the gradual maturation of bone tissue. Increasing evidence suggests that osteocytes actively modify their perilacunar matrix through PLR, involving both mineral removal and redeposition, thus altering local mineral content and crystallinity without replacement of the entire tissue volume.^1^^,^^44–50^

Furthermore, the microporosity (a function of micropetrosis-filling of lacunar space with mineral) was lower in fracturing individuals (Rokidi-2019)^51^ in bone tissue from fracturing postmenopausal women than in age- and BMD-matched controls. These data support the hypothesis that the different mechanism of mineral deposition in peri-lacunar space may be responsible for the difference in both compositional and strength properties between the T1D (Case) and Controls.

Our data (ASBMR, 2021, unpublished)^52^ from high-resolution microCT suggest that the number, density, and average volume of the lacunae decline in the bone tissue from T1D women compared to the age- and BMD-matched controls. Furthermore, in a longitudinal trans-menopausal study, we reported similar data with shrinking lacunar volume in bone tissue from women who became postmenopausal^25^ affecting their intrinsic properties. Our data from an animal-disuse study^53^ suggest shrinking lacunar volume and shape with 4 wk of tail-suspension. These data suggest that lacunar compositional properties along with morphology are influenced by health conditions (diabetes, etc.) along with disuse and therefore may affect intrinsic strength properties of bone tissue.^52^^,^^53^ Although the mechanism is still under investigation, the shrinking lacunae^52^^,^^53^ may cause greater mineralization of tissue and homogeneity, which could lead to greater brittleness and microcrack propagation in bone tissue. These compositional changes—such as higher mineral-to-matrix ratio, reduced organic content, and altered crystal maturity—are known to influence local mechanical behavior. Specifically, increased mineral content and reduced heterogeneity can enhance stiffness but reduce toughness, making the tissue more prone to microcrack initiation and propagation. These observations underscore the critical role of bone composition in determining local material properties such as elasticity, hardness, and resistance to fracture. Hence, investigation of the local peri-lacunar properties may offer new insights into mechanisms resulting in fragility fractures.

Furthermore, the direct relationship between mineral compositional (MMA, MMP) and mechanical properties (Er and H) for both Controls and T1D agrees with our earlier work.^12^ However, while both MMCA and MMCP have direct relation with Er in T1D patients, the relation between mineral maturity crystallinity (MMCA, MMCP) with Hardness is inverse—suggesting that increasing crystal size may make the bone tissue brittle at these sites, and therefore, less hardened. These data strongly suggest that localized tissue properties provide insight into the changing behavior of bone tissue with diseases and should be further investigated. Finally, we have recently reported that nanoindentation mechanical outcomes on cement lines correlate with the rate of change (slope) in material properties in the immediate vicinity of the nano indents (our T1D paper). The results of the present study agree with the previously published ones, indicating that the rate of change in material properties is an important factor in determining the mechanical attributes of the bone composite material.

## Limitations

The major limitation of the present study is the number of samples available for analysis. Obtaining samples for bone studies through biopsies can be a major challenge due to the difficulty in finding volunteers willing to undergo the bone biopsy procedure. Moreover, ensuring that the individuals fit the Control/Case requirements of the study also reduces the eligible volunteers.

The second limitation of the study involves the scan size of the Hysitron Nanoindenter. Our machine was limited to a 30 μm ×30 μm scan area, restricting the distance from which we could study a lacuna. In the future, when the device is capable of a larger scan area, it will be important to measure intrinsic material properties further than 25 μm from a lacuna.

Lastly, due to this study measuring strictly trabecular lacunae, the final limitation was the difficulty of finding lacunae in the trabecular region of bone biopsy samples. The visual density of lacunae in the trabecular bone was lower than in the cortical sections of the same samples. Nonetheless, this is a first study quantifying differences in both the compositional and intrinsic material strength properties of peri-lacunar spaces between T1D and Controls.

## Conclusion

In summary, this is the first study to explore the compositional and mechanical properties of trabecular bone in individuals with T1D by examining regions “near” (5 μm) and “each far” (25 μm) from the lacuna. Using a combination of nanoIR and Hysitron nanoindentation, the study effectively highlights the differences between “lacunae-near” and “lacunae-far” as well as between Case (T1D) and Control samples. Both the compositional properties (MMA, MMP) and the nanoindentation results reveal a significant difference (*p* < .05) in hardness & modulus values between T1D (Cases) and Controls in their “lacunae-near” and “lacunae-far” regions along with the combined Case/Control groups. While the material compositional ratio of MMCA was higher, the MMCP and hardness tended to be higher in “lacunae-near” than “lacunae-far” for all the biopsies. Our ongoing work is integrating spatially matched AFM-IR and nanoindentation measurements to examine the relationship between local composition and mechanical properties in bone biopsy tissue. By co-mapping the osteocyte peri-lacunar regions, we aim to correlate nanoscale variations in mineral and organic matrix composition with corresponding changes in mechanical strength, providing a comprehensive understanding of bone matrix quality surrounding the lacunae.

## Data Availability

The research data supporting this publication may be available upon request.
